# *Ab ovo*: Factors Affecting the Radial Stiffness of Thoracic Aorta Stent-Grafts

**DOI:** 10.17691/stm2021.13.1.02

**Published:** 2021-02-28

**Authors:** I.Yu. Zhuravleva, T.P. Timchenko, S.V. Vladimirov, M.M. Lyashenko, E.V. Kuznetsova, A.M. Chernyavskiy

**Affiliations:** Professor, Head of the Laboratory of Bioprostheses; Meshalkin National Medical Research Center, Ministry of Health of the Russian Federation, 15 Rechkunovskaya St., Novosibirsk, 630055, Russia; Junior Researcher, Laboratory of Bioprostheses; Meshalkin National Medical Research Center, Ministry of Health of the Russian Federation, 15 Rechkunovskaya St., Novosibirsk, 630055, Russia; Engineer, Laboratory of Bioprostheses; Meshalkin National Medical Research Center, Ministry of Health of the Russian Federation, 15 Rechkunovskaya St., Novosibirsk, 630055, Russia; Acting Head, Department of the Aorta and Coronary Arteries; Meshalkin National Medical Research Center, Ministry of Health of the Russian Federation, 15 Rechkunovskaya St., Novosibirsk, 630055, Russia; Research Assistant, Laboratory of Bioprostheses; Meshalkin National Medical Research Center, Ministry of Health of the Russian Federation, 15 Rechkunovskaya St., Novosibirsk, 630055, Russia; Professor, Director, Meshalkin National Medical Research Center, Ministry of Health of the Russian Federation, 15 Rechkunovskaya St., Novosibirsk, 630055, Russia

**Keywords:** hybrid aortic surgery, aortic stent-graft, radial stiffness of the stent-graft, stent oversizing, d-SINE syndrome, phase transitions of nitinol

## Abstract

**The aim of the investigation** was to study the factors influencing the radial stiffness of the thoracic aorta stent-grafts with the stent elements made of nitinol tubes by laser cutting and thermal shape setting.

**Materials and Methods.:**

The work used stent elements made by different technologies by two different manufacturers from a nitinol tube with a wall thickness of 0.5 mm (E1) and 0.4 mm (E2), with a final diameter of 20 mm. Height of cells E1 — 15 mm, E2 — 12.5 mm. The stents were manually attached to a tubular woven non-crimped base (PTGO Sever, Russia) with a 6/0 suture, resulting in either single or continuous stitches. In the RLU124 radial force tester (Blockwise Engineering LLC, USA), each of the four stent-grafts, as well as their individual stent elements, were compressed by 10 mm from the initial diameter. The dependence of the radial forces on deformation under loading and unloading was graphically presented. The temperature and enthalpy of phase transitions of nitinols into the austenite (Af) and martensitic (Mf) phases were studied using differential scanning calorimetry (DSC-3; Mettler Toledo, USA). All indicators were compared with the characteristics of two commercial models — Cronus (China) and E-vita Open Plus (Germany).

**Results.:**

Four prototypes of SibHybrid stent-grafts were tested; those differed in their stent elements, distances between them, and the type of sutures (single or continuous). The stent elements of the models studied differed in the values of Af, Mf, and the enthalpy of phase transitions of nitinols. The hardest stent was the E2 prototype. The fixation of stent elements to the woven fabric in the graft increased the radial force by 4.0–5.5 times. During compression by 50 and 20% of the original diameter, the SibHybrid models developed radial force 4.5–6.0 times greater compared with the E-vita Оpen Plus model. The radial force values of SibHybrid models were almost the same as for the Cronus and models at 20% compression. Using continuous twining round suturing increased the radial force by about 10 N; accordingly, SibHybrid E2 had the highest radial force because it was fixed by a continuous suture. The density of the stent elements fixed on the fabric did not affect the radial force of the stent-graft as a whole.

**Conclusion.:**

In the manufacture of stent elements from nitinol tubes, the main factor determining the radial stiffness is the technology of nitinol shape setting. With the standard technology of thermal shape setting, radial force can be changed by varying the height of the structure cell element and the cross-sectional area of the cell bars, as well as the suturing technique.

## Introduction

**Figure 1:**
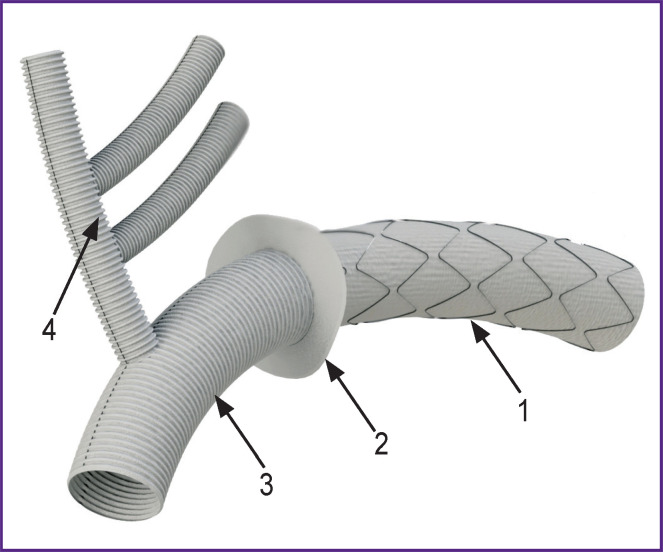
Hybrid thoracic aortic prosthesis (3D-model): (1) stent-graft for the descending part of the aorta; (2) a sealing cuff; (3) stentless crimped prosthesis of the arch and the ascending part of the aorta; (4) prosthesis can be supplemented with brachiocephalic branches

Thoracic aortic stent-grafts are, as a rule, synthetic vascular prostheses with stent elements made of nitinol and attached to the graft. They are widely used at present for endovascular correction of acute and chronic aortic dissections and also as hybrid prostheses. Hybrid aorta surgery is a relatively new trend developing after 2003 when Haverich et al. published a paper [1] describing this novel technique, which they called the “frozen elephant trunk” (FET) procedure. This technique combines minimally invasive trans-catheter implantation of stent-grafts into the descending aorta with the traditional open surgery on the arch and ascending aorta. The technique uses conventional crimped synthetic prostheses that are usually sutured to a stent-graft at the manufacturing stage (Figure 1). This approach simplifies and speeds up the operation, and also standardizes its technical aspects [2]. Hybrid prostheses can have additional branches in the area of the crimped prosthesis, for example, brachiocephalic and perfusion branches.

In the world market, there is a wide variety of stent-grafts designed for endovascular implantation, while hybrid prostheses are represented by only four models. These are two international products: E-vita Оpen Plus (JOTEC Gmbh., Germany) and Thoraflex Hybrid (Terumo Aortic, UK), and two national ones: Cronus (MicroPort, China) and J Graft Open (Japan Lifeline, Japan). All four of them differ in their design and method of fixing the stent elements. The tubular part of these stent-grafts is made of special fabrics based on polyester (polyethylene terephthalate) fibers, which differ in the weaving technique [3].

One of the most important characteristics of stent-grafts is their radial stiffness (RS), i.e. the force they apply to the wall of the true lumen of the dissected aorta. In case of insufficient RS, the stent-graft will not be held tightly in the implantation zone, which can result in its displacement and endoleaks. With an excessive RS, its distal part can break the aortic wall tissue that separates the true and false lumina, causing dislocation of the distal end of the stent-graft into a false lumen [4–6]. The latter complication is well known in the world literature as d-SINE syndrome (distal stent-graft induced new entry).

Despite the fact that the hybrid prosthesis is the top trend in thoracic aorta surgery and stimulates designing of novel stent-grafts [7–9], the question of their optimal stiffness remains open. Also, there is very little data on factors affecting RS of the entire stent. In particular, the role of various suture techniques used to fix the stent elements to the graft is completely ignored.

All stents used for commercial stent-grafts are made of wire, despite the fact that the vast majority of coronary and peripheral stents (not only nitinol ones) are made of tubular elements by laser cutting and subsequent molding. This method makes it possible to obtain more reliable products, since in their manufacture, unlike the wire-made ones, there is no need to connect the edges, which is the most weak link in the design of the structure.

**The aim of this work** was to study the factors influencing the radial stiffness of four prototypes of thoracic aortic grafts containing stents made from nitinol tubes by laser cutting and thermal shape setting, in comparison with the characteristics of two commercial stent-grafts Cronus and E-vita Оpen Plus.

## Materials and Methods

**Figure 2:**
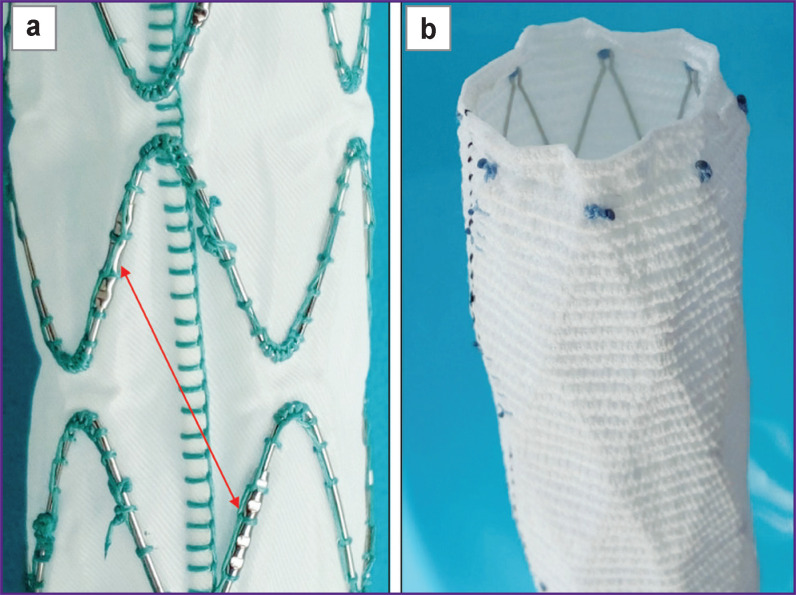
Stent-graft parts of the hybrid prostheses E-vita open Plus (a) and Cronus (b) Arrow points to sleeves connecting the edges of the wire elements

**Figure 3:**
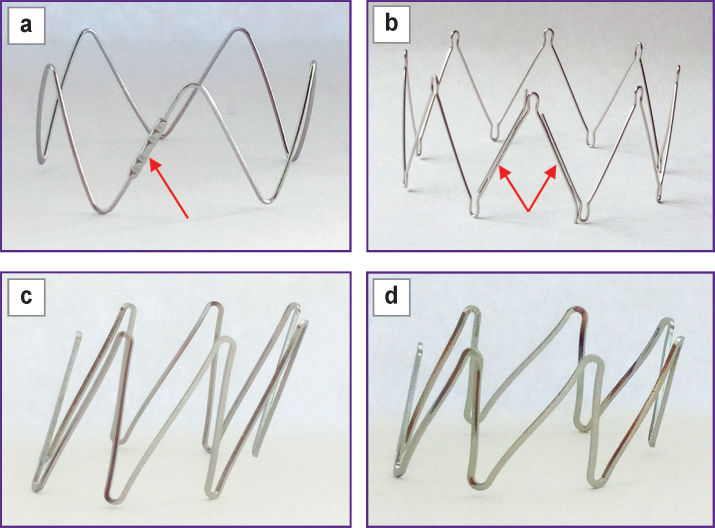
Stent elements of the hybrid prostheses E-vita open Plus (а) and Cronus (b) and the prototypes SibHybrid E1 (c) and E2 (d) Arrows indicate connections of wire elements

In the study, in addition to self-made prototypes, two commercial stent-grafts were used: a Cronus with a diameter of 28 mm and an E-vita Оpen Plus with a diameter of 19 mm (Figure 2). The stent elements of these grafts were made of nitinol wire and sutured to the graft part: in the Cronus model — from the inside, with single sutures, and in the E-vita Оpen Plus — from the outside, with a continuous loop with an overlap. In the E-vita Оpen Plus model, the edges of the wire (diameter — 0.365 mm, cross-sectional area *S_CS_* — 0.104 mm^2^) are connected by a sleeve (see Figure 2; Figure 3). The Cronus model has a wire with a diameter of 0.446 mm and *S_CS_* of 0.156 mm^2^; the edges of the wire elements are elongated and connected by sutures (see Figure 3).

It should be noted that the Cronus stent element (free of fabric) has an internal diameter of 42 mm. The diameter of the E-vita Оpen Plus stent is also larger than the stent-graft diameter by 3 mm and reaches 22 mm. Therefore, these stents are fixed to the graft in a stress-strain state.

### Prototype manufacturing.

In the manufacture of the four original prototypes, which received the working name SibHybrid (SH), we used non-crimped woven vascular prostheses with a diameter of 20 mm (PTGO Sever, Russia) and stent elements E1 and E2, manufactured by two different (domestic and foreign) manufacturers according to our technical specifications and our drawings (Table 1; see Figure 3). Both types of elements were laser cut from a nitinol tube, followed by their shaping by heat treatment and by final electro-polishing. The elements have a complex spatial shape of a cylinder, the bases of which are formed by secant planes directed at an angle of 50° to its axis (Figure 3 (c), (d)). When fixed to fabric, such stent elements resemble a Z-shaped helix.

**Table 1:** Parameters of stent elements used for the production of SibHybrid stent-grafts prototypes

Parameter	E1	E2
Manufacturer	Domestic	Foreign
Tube outer diameter (mm)	7.0	7.0
Tube wall thickness (mm)	0.50	0.40
Sectional area of the cell beam (mm^2^)	0.25	0.20
Number of cells	7	7
Inner diameter of the stent element (mm)	20.0	20.0
Cell type	Open	Open
Development drawing	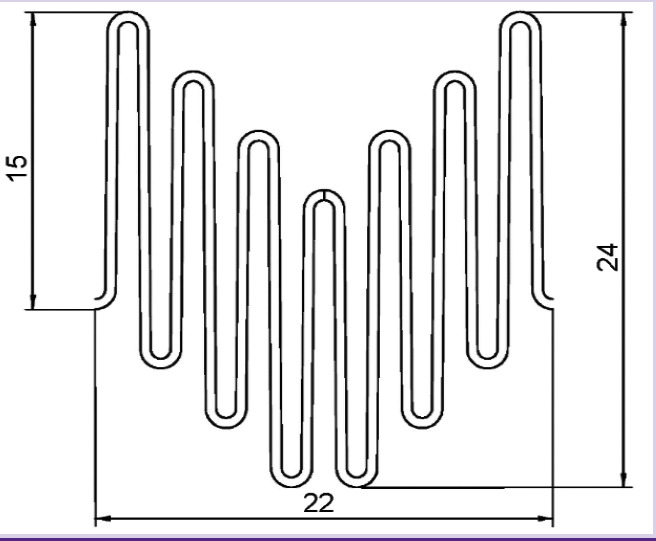	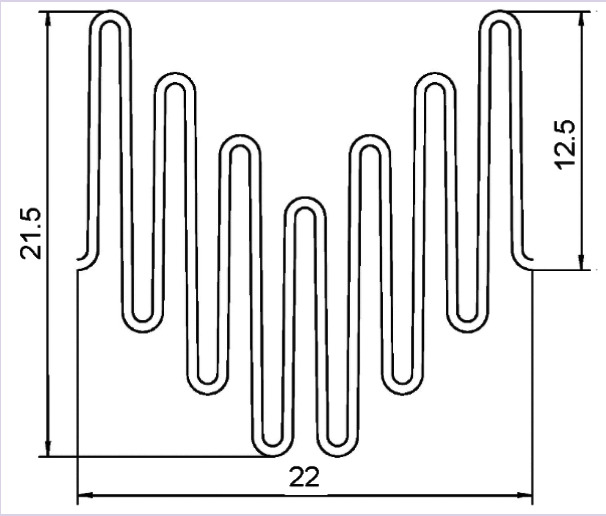

The stent elements were manually fixed to the fabric base from the outer surface using single sutures or continuous sutures from Premicron 6/0 (B. Braun, Germany).

**Figure 4:**
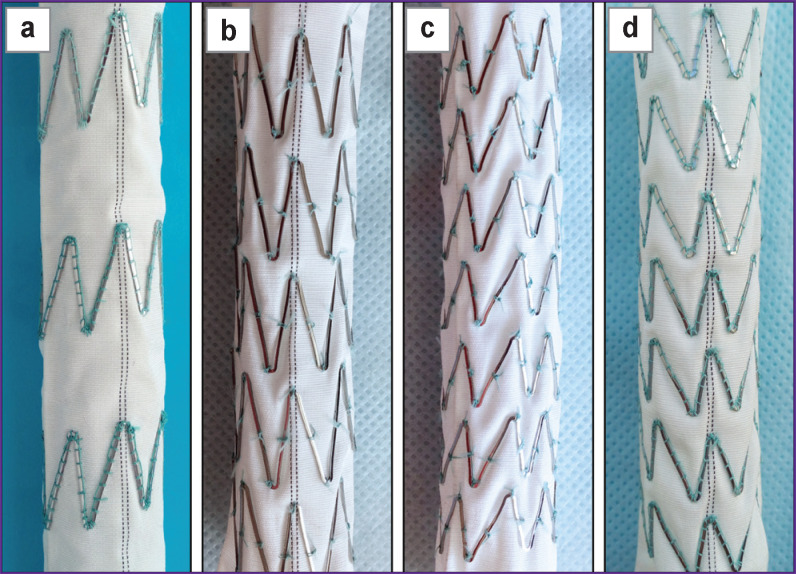
Prototypes of SibHybrid stent-grafts: (a) SH1; (b) SH2; (c) SH3; (d) SH4

The characteristics of the four SH prototype stent-grafts are presented in Table 2 and Figure 4.

**Table 2:** Characteristics of the SibHybrid prototypes

Characteristic	SH1	SH2	SH3	SH4
Distance between the cell apices (mm)	35	18	13	13
Stent element	E1	E1	E2	E2
Type of sutures	Continuous	Single	Single	Continuous

### Radial stiffness tests.

**Figure 5:**
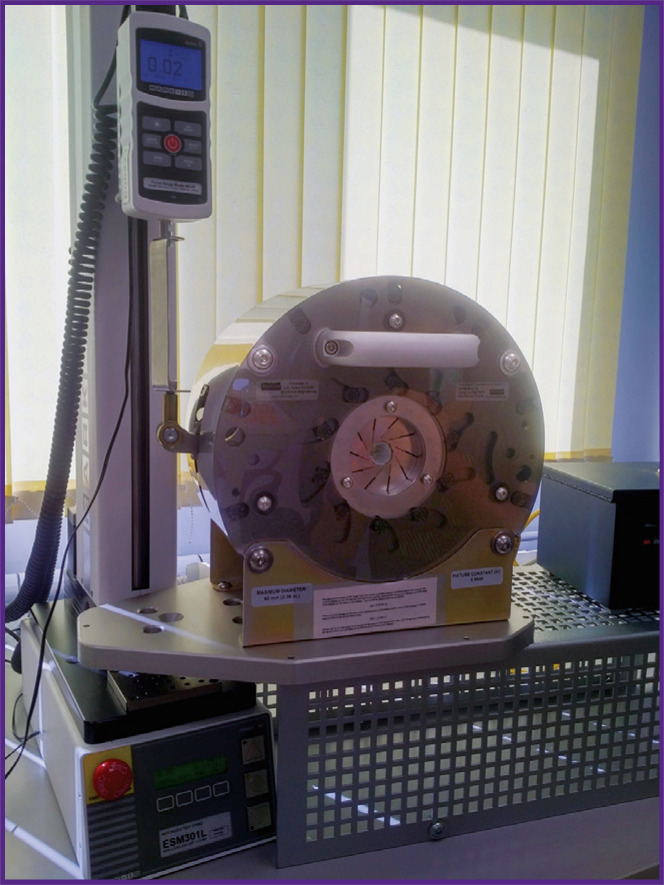
Radial force tester RLU 124 (Blockwise Engineering LLC, USA)

The radial forces of the stent-grafts were studied using a radial force tester RLU124 (Blockwise Engineering LLC, USA) (Figure 5); in the device, the plates compressed the tested structures at a rate of 10 mm/min along the long axis so that the initial diameter of the stent-graft changed by 10 mm.

Together with the final stent-graft, we tested individual stent elements E1, E2, and the elements of both commercial models. Since the Cronus was compressed to 18 mm (10 mm change from the original 28 mm diameter), its stent element was also compressed to 18 mm.

The testing procedure was carried out at temperatures of 24–26°С and 37°С in 3–4 repeats at each temperature. Measurements of radial forces under load–unload modes was performed using an ESM301L universal testing machine (Mark-10 Corporation, USA). The results were exported to Microsoft Excel, and each point was recalculated according to the formulas recommended by the RLU124 manufacturer:

TRF=F⋅2.099,

where *TRF* is the radial forces developed by the test object, *F* are the forces as measured by the tester;

ΔD=ΔX⋅0.9525,

where Δ*D* is the change in the diameter of the tested object, and Δ*x* is the movement as measured by the measuring device.

The data obtained from all measurements were used to plot the force-strain graphs.

### Determination of the phase transition temperature of nitinol stent elements.

**Figure 6:**
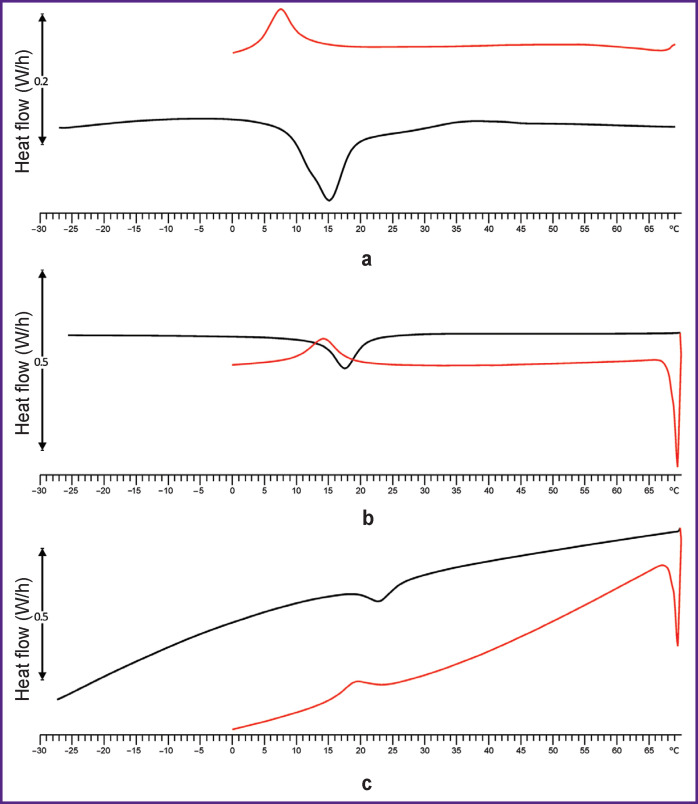
Typical curves obtained by differential scanning calorimetry of stents E1 (a), E2 (b), and E-vita Оpen Plus (c)

The temperature of phase transitions of nitinol from stent elements was studied using a DSC-3 differential scanning calorimeter (Mettler Toledo, USA). For this, three straight 5 mm long segments were cut out from the stent elements of all stent-grafts. Each sample was cooled to –30°C and then heated to +70°C at a rate of 10°C/min. Based on the final point of the endothermal peak in the heating curve, the temperatures of the start (*T_onset_*) and the end (*T_endset_*) of the nitinol transition from the martensitic (Mf) to austenitic (Af) state were determined (Figure 6). The enthalpy of the phase transition (Δ*H*) was calculated automatically by the device based on the peak area and sample mass. After that, the test object was cooled again, and the end temperature of the transition from the austenitic to martensitic state was recorded.

The results of these experiments were **statistically processed** using the Statistica 13.0 software (Dell Software Inc., USA). Results were expressed as mean ± standard deviation (M±σ). The normal distribution in each group was verified using the Shapiro–Wilk test. Since the data distribution corresponded to the normal one, the significance of the differences was calculated by the Student’s t-test, considering the differences statistically significant at p<0.05.

## Results

One of the important parameters that determine the stent stiffness is the material itself, i.e. nitinol, and the way it had been thermally treated. These factors have an impact on the temperature of phase transition from the martensitic to austenitic state of nitinol, and can also modify the enthalpy of this transition. The temperatures where the nitinols E1, E2, and E-vita Оpen Plus begin and complete their transition into the austenite phase are shown in Table 3 together with the enthalpy of the transition; the end temperature of transition into the martensitic phase is shown as well.

**Table 3:** Differential scanning calorimetry of the stent elements (M±σ)

Phase	Index	Test object			
			E1 (1)	E2 (2)	E-vita Open Plus (3)
Af	*T_onset_* (°С)		8.88±0.0	14.41±0.21	18.84±0.65
	p	(1)		0.0000	0.0000
		(2)	0.0000		0.0000
		(3)	0.0000	0.0000	
	*T_endset_* (°С)		18.33±0.52	21.02±0.45	25.94±0.41
	p	(1)		0.0000	0.0000
		(2)	0.0000		0.0000
		(3)	0.0000	0.0000	
	–Δ*H* (J/g)		3.45±0.25	1.97±0.12	1.77±0.45
	p	(1)		0.0000	0.0000
		(2)	0.0000		**0.2052**
		(3)	0.0000	**0.2052**	
Mf	*T_endset_* (°С)		4.33±0.09	10.51±0.18	15.34±0.31
	p	(1)		0.0000	0.0000
		(2)	0.0000		0.0000
		(3)	0.0000	0.0000	

In this experimentation, we encountered unexpected effects when testing the Cronus model nitinol: in the range of temperatures commonly used to test medical nitinols, we found no phase transition peaks. Therefore, we extended the temperature range and scanned from –150°С to +450°С; then we detect a peak in the range from –120°С to –140°С.

**Figure 7:**
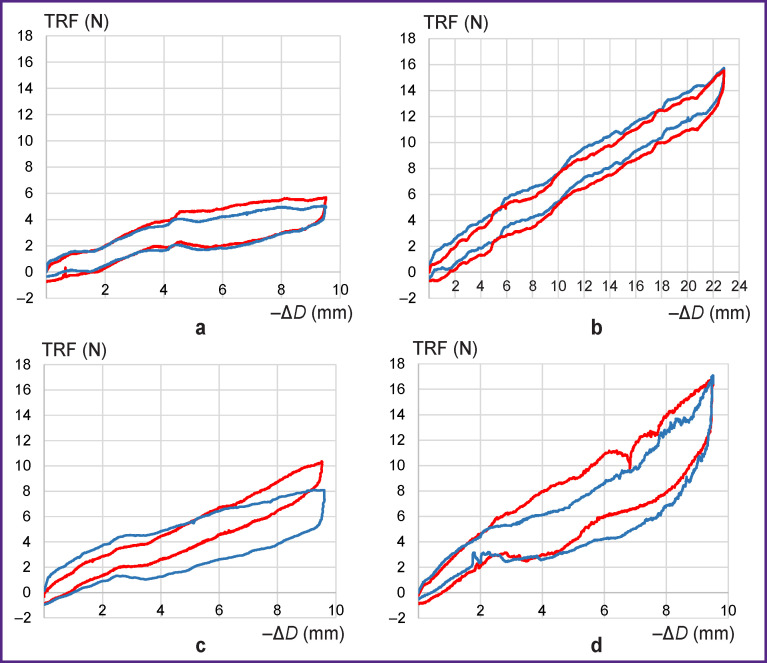
Load-unload curves reflecting the deformation of stent elements: (a) E-vita Оpen Plus; (b) Cronus; (c) E1; (d) E2. Blue line — at 24**–**26°С; red line — at 37°С

When testing the RS of individual stent elements, the following patterns were revealed (Figure 7):

1. In all tested groups, the load–unload curves did not depend (or depended only a little) on the temperature at which the procedure was carried out. This effect is not surprising since even at 24–26°C, stents E1, E2, and E-vita Оpen Plus are already in the austenitic state and their mechanical properties should not be fundamentally changed when the temperature rises to 37^o^C.
2. E2 stents are known for the highest RS (about 17 N) when compressed by 50% of the initial diameter (i.e. by 10 mm). For E1, this RS value is about 10 N, despite the fact that the *S_CS_* of the cell bar in E1 is 25% higher than that in E2. At 50% compression (up to 21 mm), the Cronus stent element develops a force of 14 N and the E-vita Open Plus — about 6 N.

**Figure 8:**
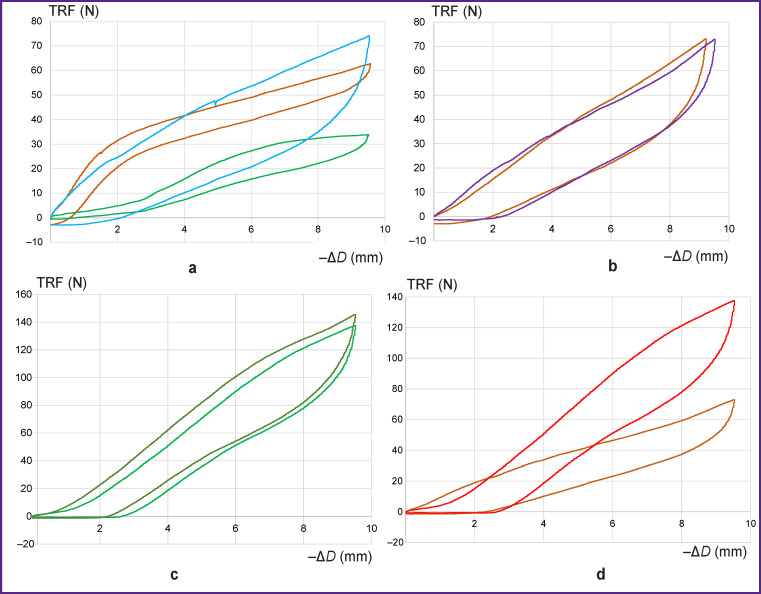
Load-unload curves reflecting the compression of stent-grafts (stent elements + fabric) over 10 mm. Test temperature — 37°С: (a) *brown* — Cronus, *green* — E-vita Оpen Plus, *blue* — SH1 (influence of the design as a whole); (b) *brown* — SH1 (“rare” stents), *violet* — SH2 (“frequent” stents); (c) *green* — SH3 (single sutures), *dark green* — SH4 (continuous sutures); (d) *brown* — SH2 (stent E1, single sutures), *red* — SH3 (stent E2, single sutures)

After attaching the stent elements to the fabric, the radial force increases sharply (Figure 8 (a)): at the end point of compression, it was 34 N for the E-vita Open Plus model, 63 N for Cronus, and 74 N for our SH1.

Quite unexpected was the fact that for the SibHybrid models, the density of stent elements fixed on the fabric had almost no effect on the RS of the stent-graft as a whole (Figure 8 (b)). Thus, the load–unload curve for SH2 practically did not differ from that for SH1, although SH2 contained twice as many stent elements.

The effect of the fixation technique on the RS of stent-grafts is shown in Figure 8 (c). Over the longer part of the load curve, the values of radial forces are approximately 10 N higher for SH4 compared to SH3; the two models differ only by the suturing mode: continuous — in SH4 and single — in SH3.

Obviously, the main role in the RS differences between various grafts is played by the stent elements (Figure 8 (d)). Samples SH3 and SH2, having the same diameter, fixed to the same woven base with the similar sutures at equal intervals between the elements, still have different stiffness values: at the end point of compression, it is almost 140 N for SH3 that contained the stiffest E2 vs 73 N for SH2, containing E1 stents.

## Discussion

The clinical aspect of stent-graft “over-stiffness” is different from the bioengineering consideration. For the latter, excessive stiffness is associated with the mechanical incompatibility of the implants with the pathologically altered aortic wall. Surgeons, however, tend to attribute d-SINE to individual variability in the conical shape of the aorta, which, in fact, is the normal anatomical difference in diameters between the proximal and distal parts of the aorta [4]. Indeed, as the diameter of the aorta decreases in the distal direction, the radial forces developed by the stent-graft increase because the graft diameter remains unchanged but the aorta lumen narrows. Jang et al. [10] proposed to calculate the “oversizing coefficient” (excess of the stent-graft diameter over the diameter of the implantation zone) based on the size of the true lumen of the dissected aorta in the distal zone and the distal size of the implanted stent, showing that oversizing more than 20% is a reliable predictor of d-SINE.

Based on our results, an analogue of clinical oversizing can be proposed. This is expressed as compression of the stent-graft by 20% of the initial diameter, i.e. 4 mm for E-vita Оpen Plus and other models of the SibHybrid line, and 5.6 mm — for the Cronus models. In Figure 8 (a) and (c), it is clearly seen that when compressed by 20%, the E-vita Оpen Plus develops radial forces of 16 N, SH1 — 41 N, Cronus — 47.5 N, SH3 with single suturing — 50.5 N, and a similar SH4 with running sutures — 62 N. Thus, with the same oversizing of 20%, an almost 4-fold difference in radial forces can be observed between the “softest” and “hardest” stent-grafts tested in this study. The question remains though, what model is preferable.

It is impossible to answer this question while having no information on the mechanical characteristics of the implantation zone, i.e. the walls of the diseased aorta (especially under dissection). In the literature, there are few reports on this issue [11–17]; those mainly concern the properties of the aortic wall with an aneurysm, but not with dissection. However, without knowing what loads the weakened walls of the dissected aorta can withstand, we cannot determine what should be the values of stiffness and oversizing for the potential stent-graft. Notably, these values can easily be changed.

In this study, we have shown how the stiffness of stent-grafts is influenced by the design and material of the stent element, as well as the fixation technique (continuous suturing increases the stiffness by more than 20%). At the same time, the number of stent elements sutured to the fabric (stent density) has practically no effect on the stiffness of the stent-graft. Considering how sharply the RS of the stent-graft increases in comparison with the RS of an individual element, we assume that the characteristics of the fabric itself (thickness and composition of the fiber, weaving rapport, etc.), as well as the properties of the sealing impregnation, can affect the stiffness of SH products. In this work, we did not aim to study this issue, but it is part of our future research.

With regard to the design of the wire stent, it is known that its stiffness increases with an increase in the cross-sectional area *S_CS_* and a decrease in the height of the structure cells when their shape and number remain unchanged [18]. Based on our data for stents made from a tubular blank, we consider the height of the cell and the method of nitinol thermal shape setting to be factors of the major significance. Thus, in SH1 and SH2, elements with *S_CS_* 0.25 mm^2^ and a height of 15 mm were used; those were much softer than SH3 and SH4 with cells 12.5 mm high and *S_CS_* 0.20 mm^2^. Although for wire stents, this relationship may be somewhat different, but it can be modified by varying the height and *S_CS_* of the structure cells. No doubt, the method of thermal treatment for nitinol affects the stiffness of such stents [19]. Our E1 and E2 models were made by two different manufacturers using different shaping technologies. With differential scanning calorimetry, we were able to reveal differences in phase transition temperatures and enthalpy between these two products, thus emphasizing different properties of these two materials.

It should be noted that when testing grafts of different designs, it is important to test the entire range of similar grafts (of the same standard sizes) for radial compression, without extrapolating the results from one diameter to other. While testing circular stent elements used in Thoraflex Hybrid and Anaconda (Terumo Aortic) models, Senf et al. [20] showed that with the same oversizing value of 20%, an increase in the stent diameter by 1.7-fold led to an increase in its radial forces by 1.5-fold, for which the authors could not find a valid explanation.

It should be noted that manufacturers of both endovascular and hybrid stent-grafts do not disclose the specifics of their RS measurements, although this characteristic is of key importance for these devices and largely determines the quality of clinical results. We consider this practice to be flawed, as the risks of complications are ultimately borne by surgeons and patients. A typical example is the clinical experience of using the Thoraflex Hybrid prosthesis, the stent-graft of which was originally conceived as “soft”; it was also perceived as soft by surgeons according to tactile sensations. Subsequently, this stent-graft turned out to be more rigid than the E-vita Оpen Plus [5] and caused the development of d-SINE more often [6].

Thus, the study of the first samples of aortic stent-grafts, the stents of which were made from nitinol tubes by laser cutting and thermal shaping, showed that they had a significantly higher RS at *S_CS_* of a cell bar comparable to *S_CS_* of wire nitinol stents. At the same time, the density of the stent elements fixed on the fabric does not affect the stiffness of the stent-graft structure; changes in the suturing technique from single to continuous sutures increase the stiffness by an average of 10 N. Differences in nitinol thermal shaping technologies may cause significant differences in the stiffness of the stent elements; the thermal characteristics of the materials can be partially revealed with differential scanning calorimetry.

Therefore, varying the tube thickness, cell bar width and height, as well as the type of fixing sutures can be used for achieving optimal RS only after standardization of the thermal treatment method by the manufacturer of the stent elements. In this case, differential scanning calorimetry can serve as a quality control method. However, the question of optimal values of RS itself remains open and requires large-scale studies on mechanical properties of the diseased aorta.

The limitations of this work include the lack of data on nitinol processing technologies for SibHybrid prototypes. According to the confidentiality agreement signed by the authors, they are not allowed to disclose information about the manufacturers and manufacturing technologies of stent elements. In addition, the effect of different woven bases and hermetical sealing on the RS of stent-grafts is yet to be studied in our future research.

## Conclusion

In the manufacture of stent elements of stent-grafts from nitinol tubes, the main factor determining the radial stiffness is the technology of nitinol thermal shaping. With the standardized shaping technology, the stiffness can be controlled by changing the height of the cell and the cross-sectional area of its bars, as well as by suturing technique.
